# Auranofin is active against *Histoplasma capsulatum* and reduces the expression of virulence-related genes

**DOI:** 10.1371/journal.pntd.0012586

**Published:** 2024-10-07

**Authors:** Marcos de Abreu Almeida, Lilian Cristiane Baeza, Leandro B. R. Silva, Andréa Reis Bernardes-Engemann, Fernando Almeida-Silva, Rowena Alves Coelho, Iara Bastos de Andrade, Dario Corrêa-Junior, Susana Frases, Rosely Maria Zancopé-Oliveira, Alexandre Alanio, Carlos Pelleschi Taborda, Rodrigo Almeida-Paes

**Affiliations:** 1 Laboratório de Micologia, Instituto Nacional de Infectologia Evandro Chagas, Fundação Oswaldo Cruz, Rio de Janeiro, Brazil; 2 Departamento de Microbiologia e Parasitologia, Instituto Biomédico, Universidade Federal Fluminense, Niterói, Brazil; 3 Centro de Ciências Médicas e Farmacêuticas, Universidade Estadual do Oeste do Paraná, Cascavel, Brazil; 4 Departamento de Microbiologia, Instituto de Ciências Biomédicas, Universidade de São Paulo, São Paulo, Brazil; 5 Department of Microbiology and Immunology, The University of British Columbia, Vancouver, Canada; 6 Laboratório de Biofísica de Fungos, Instituto de Biofísica Carlos Chagas Filho, Universidade Federal do Rio de Janeiro, Rio de Janeiro, Brazil; 7 Rede Micologia, FAPERJ, Rio de Janeiro, Brazil; 8 Institut Pasteur, Université Paris Cité, Centre National de Référence Mycoses Invasives et Antifongiques, Groupe de recherche Mycologie Translationnelle, Département de Mycologie, Paris, France; Universidad de Antioquia, COLOMBIA

## Abstract

**Background:**

Auranofin is an approved anti-rheumatic drug that has a broad-range inhibitory action against several microorganisms, including human pathogenic fungi. The auranofin activity against *Histoplasma capsulatum*, the dimorphic fungus that causes histoplasmosis, has not been properly addressed. Since there are few therapeutic options for this life-threatening systemic mycosis, this study evaluated the effects of auranofin on *H*. *capsulatum* growth and expression of virulence factors.

**Methodology/principal findings:**

Minimal inhibitory and fungicidal concentrations (MIC and MFC, respectively) of auranofin against 15 *H*. *capsulatum* strains with distinct genetic backgrounds were determined using the yeast form of the fungus and a microdilution protocol. Auranofin activity was also assessed on a macrophage model of infection and on a *Tenebrio molitor* invertebrate animal model. Expression of virulence-related genes was compared between auranofin treated and untreated *H*. *capsulatum* yeast cells using a quantitative PCR assay. Auranofin affected the growth of different strains of *H*. *capsulatum*, with MIC and MFC values ranging from 1.25 to 5.0 μM and from 2.5 to >10 μM, respectively. Auranofin was able to kill intracellular *H*. *capsulatum* yeast cells and conferred protection against the fungus in the experimental animal model of infection. Moreover, the expression of catalase A, HSP70, superoxide dismutase, thioredoxin reductase, serine proteinase, cytochrome C peroxidase, histone 2B, formamidase, metallopeptidase, Y20 and YPS3 proteins were reduced after six hours of auranofin treatment. CONCLUSIONS/SIGNIFICANCE: Auranofin is fungicidal against *H*. *capsulatum* and reduces the expression of several virulence-related genes, which makes this anti-rheumatic drug a good candidate for new medicines against histoplasmosis.

## Author summary

Histoplasmosis is a serious disease caused by the fungus *Histoplasma capsulatum*, which can be life-threatening and has limited treatment options. In this study, we investigated the potential of auranofin, a drug originally approved for treating rheumatoid arthritis, to combat *H*. *capsulatum*. We tested auranofin against different strains of the fungus and found that it effectively inhibited fungal growth. This drug was also effective in killing the fungus within cells from the immune system and provided protection in an experimental animal model. Furthermore, auranofin reduced the expression of several genes associated with the fungus’s ability to cause disease. These genes are involved in critical functions such as detoxifying harmful substances and maintaining cellular structures. By suppressing these genes, auranofin hampers the fungus’s ability to survive and cause infection. These findings suggest that auranofin could be repurposed as a treatment for histoplasmosis, offering a new avenue for therapy against this challenging infection. This research is significant because it highlights the potential of existing drugs to treat other diseases, broadening our arsenal of available treatments and contributing to better healthcare solutions.

## 1 Introduction

Auranofin is a gold-containing orally administered approved drug used for more than 40 years to treat rheumatoid arthritis. However, since the introduction of biological therapies for this disease, such as the chimeric monoclonal antibody infliximab, the original use of auranofin has remarkedly decreased. Due to the auranofin safety, guaranteed by its four decades of use with low contraindications, this drug became an interesting target for drug repurposing [1]. In fact, studies, in different stages of clinical research, are in development to repurpose auranofin to several diseases, infectious or not [2–4].

Several studies have demonstrated the broad-range antimicrobial activity of auranofin against several bacteria [5], protozoa [3,6], viruses [7] and fungi [8]. The list of auranofin-susceptible fungi includes certain *Candida* species, *Cryptococcus neoformans*, *Blastomyces dermatitidis*, *Aspergillus fumigatus*, *Paecylomyces variotii*, *Scedosporium apiospermum*, *Lomentospora prolificans*, *Saccharomyces cerevisiae* and several chromoblastomycosis agents such as *Fonsecaea pedrosoi* and *Cladophialophora carrionii* [8–10]. Despite the great auranofin antifungal properties, its activity against some important human pathogenic fungi remains unelucidated.

*Histoplasma capsulatum* is the etiological agent of histoplasmosis, a systemic mycosis that is often related to outbreaks, usually reported in caves, mines, chicken coops and abandoned places [11]. This mycosis can affect immunocompetent people, usually as a chronic pulmonary disease [12], and immunosuppressed patients, especially people living with HIV/AIDS, who develop a life-threatening disseminated disease [13]. The infection starts with the inhalation of mycelial propagules, especially microconidia, which convert to the yeast form inside or outside phagocytes present in the lungs. These phagocytes may be vehicles for fungal dissemination into the host, since the *H*. *capsulatum* yeast form is highly adapted to mammalian hosts [14]. Histoplasmosis is a prevalent opportunistic infection among people living with HIV in the Latin America region, as indicated by various studies [15–17].

A proper management of histoplasmosis depends on rapid diagnosis and specific treatment [18]. Currently, liposomal amphotericin B is the first-choice induction therapy for disseminated and moderate-to-severe histoplasmosis, followed by a consolidation regimen using itraconazole. Itraconazole alone is also the alternative for mild infections [19]. These two antifungal drugs present a series of adverse effects and drug interactions [20,21], which is a serious problem in critically ill immunosuppressed patients who receive several medications.

The antifungal pipeline includes three potential new antifungal drugs with antifungal activity against *H*. *capsulatum*: fosmanogepix, a drug that affects trafficking and anchoring mannoproteins to the fungal cell membrane and wall; ibrexafungerp, a triterpenoid that inhibits glucan synthase; and olorofim, an orotomide that targets pyrimidine synthesis. However, none of them have undergone specific clinical trials for histoplasmosis [22]. In this scenario, drug repurposing is an interesting alternative, since drugs with proven administration safety may skip some stages of clinical research [23]. As auranofin administration is safe [1] and it demonstrated antifungal activity against many fungi [24], we studied whether this anti-rheumatic drug can affect *H*. *capsulatum* growth and expression of genes that enhance fungal virulence during infection.

## 2 Material and methods

### 2.1 Strains

The reference strain *H*. *capsulatum* G217B (ATCC 26032) was used throughout the study. In addition, other 14 *H*. *capsulatum* strains, with different genotypes determined by MLST analyses in previous publications [25–28], were included in the *in vitro* antifungal susceptibility studies. All strains are maintained at the Pathogenic Fungal Collection of Fiocruz (WDCM 951). Fungal strains were maintained in the yeast-like form on ML-Gema agar medium [29] at 37°C.

### 2.2 *In vitro* antifungal tests

The minimal inhibitory concentration (MIC) of auranofin against the *H*. *capsulatum* strains was verified through the broth microdilution method according to the European Committee on Antimicrobial Susceptibility Testing (EUCAST) guideline [30], with a few modifications to test this dimorphic fungus and the antirheumatic drug, which are described herein: two-fold serial dilutions of a stock auranofin solution in dimethyl sulfoxide (2 mM) were made to obtain the final testing concentrations (range: 10 to 0.02 μM) in RPMI-1640 medium (Sigma-Aldrich Co., St. Louis, MO, USA), supplemented with 2% glucose (Neon Química, Susano, Brazil) and buffered (pH 7.2) with 3-N-morpholinopropanesulfonic acid (Sigma-Aldrich Co.). Fungal suspensions (1 × 10^6^ yeast-like cells/mL) were prepared in sterile 0.9% saline solution after five days of growth. Fungal suspensions were then added to the wells of a flat-bottom 96-well plate (Corning, Glendale, AZ, USA) containing the different auranofin concentrations. For each strain, a drug-free and a cell-free control wells were included, as positive and negative growth controls, respectively. Plates were incubated at 35°C for five days. The MIC was determined as the lowest auranofin concentration that completely inhibited fungal growth (100% inhibition), as the EUCAST method suggest for amphotericin B. Additionally, to determine the minimal fungicidal concentration (MFC) as previously published for auranofin and fungal cells [9], 5 μL from each well without visual fungal growth from the plates used for MIC determinations and incubated at 37°C were plated on potato dextrose agar (Beckton, Dickinson and Company, Sparks, MD, USA) and incubated for 21 days at 25°C. The lowest auranofin concentration without *H*. *capsulatum* growth after this incubation was defined as the MFC. Interpretations whether auranofin is fungicidal or fungistatic was as follows: if the MFC/MIC ratio was 1 or 2, it was considered fungicidal, otherwise, it was classified as fungistatic [31]. MIC and MFC determinations were performed in triplicate.

### 2.3 Synergism studies

The synergistic activity of auranofin with conventional antifungal agents was verified using the checkerboard method [32]. In this method, two drugs were loaded into a single 96-well plate, with varying concentrations of the auranofin-antifungal combination in each well. Initially, this experiment was conducted using the G217B reference strain to evaluate the synergistic effects of the combinations between auranofin and the antifungal drugs amphotericin B, itraconazole, and caspofungin (all from Sigma-Aldrich Co.). Subsequently, the experiment was replicated for the drug combinations that presented synergism using representative strains from different *H*. *capsulatum* phylogenetic species. The auranofin and antifungal dilutions were prepared following the methodology proposed by the European Committee on Antimicrobial Susceptibility Testing (EUCAST), starting from a 100-fold concentrated stock auranofin and antifungal solution [30]. The final concentrations of the auranofin and antifungal drug ranged from 0.156 to 10 μM and 0.0004 to 2 μM, respectively. The fungal inoculum and incubation conditions remained the same as those described for the *in vitro* antifungal tests. The interaction between the drugs was classified using the fractional inhibitory concentration index (FICI) [33]. The types of interactions between the drug combinations were classified as synergism if FICI ≤ 0.5, indifference if 0.5 < FICI < 4, and antagonism if FICI ≥ 4 [32,33].

### 2.4 Morphology studies

The *H*. *capsulatum* G217B yeast-like cells were treated with a subinhibitory auranofin concentration (1/2 MIC) in HAM’s F12 medium (Gibco, Grand Island, NY, USA) and incubated at 37°C for three days. A control culture without auranofin was made under the same conditions. Cells were harvested, washed three times with phosphate-buffered saline (PBS) and visualized with an AXIO Lab.A1 bright-field light microscope (Zeiss, Jena, Germany). The diameter of 100 cells of each condition was measured using the ImageJ 1.40 g software (National Institutes of Health, Bethesda, MD, USA). Three replicates were performed for each condition.

### 2.5 Regulation of gene expression

We treated *H*. *capsulatum* G217B yeast-like cells (1 × 10^6^ cells/mL) with a subinhibitory concentration (1/2 MIC) of auranofin in HAM’s F12 medium for three and six hours. The cells were harvested and subjected to total RNA extraction by mechanical cell rupture using a BeadBeater (BioSpec, Bartlesville, OK, United States), 0.5 μm diameter glass beads and TRIzol (Invitrogen, Waltham, MA, USA), according to the manufacturer’s protocol. Equal amounts of RNA (1 μg) of the RNAs were used to synthesize single stranded cDNAs (DNA complementary) using the High Capacity RNA-to-cDNA kit (Applied Biosystems, Foster City, CA) and oligo (dT) primer. Prior to PCR reactions, the cDNAs were diluted (1:5) by adding water. Quantitative PCR (qRT-PCR) was performed using the SYBR green PCR master mix (Applied Biosystems, Foster City, CA, United States), in the AriaMx real-time PCR system (Agilent Technologies, Santa Clara, CA, USA), with 10 pmol of each specific primer and 4 μL of template cDNA in a final volume of 20 μl. The genes encoding the virulence-related proteins catalase A (HCAG_05109), catalase B (HCAG_08064), heat shock protein (HSP) 70 (HCAG_01398), superoxide dismutase (HCAG_01543), thioredoxin reductase (HCAG_07019), serine proteinase (HCAG_00635), cytochrome C peroxidase (HCAG_08658), Histone 2B (HCAG_03525), formamidase (HCAG_08831), metallopeptidase (HCAG_04252), Y20 protein (HCAG_04745), and yeast phase specific protein 3 (YPS 3) (Q00950) were selected for analysis. The constitutively expressed *glyceraldehyde 3-phosphate dehydrogenase* (HCAG_04910) gene [34–36] was selected to normalize the samples. Intron spanning primers were designed using the Primer 3 tool and are listed at the S1 Table. A cDNA aliquot from each sample diluted serially at 1:5 was mixed and used to generate a relative standard curve. All analyses were performed in triplicate. The relative expression levels of selected genes were obtained using the standard curve method for relative quantification [37]. Transcription levels of cells treated with auranofin were normalized in relation to transcription levels of the untreated control condition.

### 2.6 Intracellular antifungal activity

The intracellular activity of auranofin on the *H*. *capsulatum* reference strain was determined by counting colony-forming units (CFUs) recovered from macrophage infection. J774 1.6 macrophages (Rio de Janeiro Cell Bank–BCRJ/UFRJ, accession number 0273) were employed. Macrophages (less than 10 passages) were maintained in RPMI 1640 medium (Sigma-Aldrich) with 10% v/v fetal bovine serum and MEM non-essential amino acid solution (Sigma Aldrich), at 37°C in a CO_2_ incubator (ThermoFisher Scientific, Waltham, MA, USA) until the cells were confluent. A total of 10^6^ J774 macrophages were plated per well of a 96-well plate in RPMI medium containing IFN-γ (1 U/mL) (Sigma Aldrich) and incubated for 24 h at 37°C and 5% CO_2_. In parallel, *H*. *capsulatum* yeast cells were grown in HAM’s F12 medium for 72 h. Next, 5 × 10^6^ yeast-like cells were added per well, to give a yeast:macrophage cell ratio of 5:1. After two hours of yeast/macrophage interaction, non-phagocytosed/non-adhered yeast-like cells were removed after three washes with PBS [38]. In addition, auranofin was added to the wells at the following concentrations: ¼ MIC, MIC, and 4× MIC. Controls consisted in: (i) culture medium only (sterility control); (ii) macrophages only (macrophage control); (iii) *H*. *capsulatum* yeast-like cells only (planktonic control); and (iv) *H*. *capsulatum* yeast-like cells with macrophages (5:1 ratio) without auranofin (intracellular control). Cultures were incubated for 24 h at 37°C and 5% CO_2_. After this incubation, macrophages were lysed with cold water and fungal cells were recovered. The number of viable *H*. *capsulatum* cells was determined based on CFUs counting. The lysates were cultured in ML-Gema agar medium and CFUs were determined after growth at 37°C for 10 days. Four technical replicates were performed for three biological replicates.

### 2.7 Invertebrate model for *in vivo* activity

For *in vivo* activity studies, we adapted a previously published invertebrate model [39], using *Tenebrio molitor* larvae of at least 1 cm, with regular motility, without dark spots or gray marks. Ten animals were inoculated in the last left proleg with 1 × 10^4^ *H*. *capsulatum* yeast-like cells. Animals were treated with an injection of 10 μL of a 5.7 mg/kg auranofin solution, two and 24 hours after fungal injection (auranofin group). Other animal groups consisted in: (i) non-injected larvae (sham group); (ii) traumatized with the syringe (trauma group); (iii) injected with sterile PBS (non-infected group); (iv) infected with *H*. *capsulatum* and treated with PBS (infected group); (v) infected with *H*. *capsulatum* and treated with itraconazole (Sigma-Aldrich) at the same doses and times (itraconazole group); and (vi) injected with PBS, instead of *H*. *capsulatum* cells and treated with auranofin (auranofin control group). Drug concentrations in the experiments were based a safe itraconazole dose to treat *Galleria mellonella* larvae, previously described [40]. Then, the animals were kept in Petri dishes at room temperature and were fed ad libitum on bran flour. Mortality and phenotypic changes were recorded for 14 days. Larvae that convert to pupa were censored. This experiment was performed in three biological replicates.

### 2.8 Statistical analyses

The Prism 9.0 software (GraphPad software, La Jolla, CA, USA) was used for data analyses. The normality of the data was evaluated using the Shapiro-Wilk test to decide between parametric or non-parametric tests. The comparison of cellular diameter was performed using the Student´s t test. qPCR data was evaluated using the Student’s t test. Intracellular antifungal activity was evaluated with the Wilcoxon test. Survival curves were compared using the Log-rank (Mantel-Cox) test. A 0.05 significance level was adopted for all analyses.

## 3 Results

### 3.1 Auranofin has anti-*Histoplasma* activity

Table 1 presents MIC and MFC data for the *in vitro* auranofin susceptibility test of the 15 *H*. *capsulatum* strains. Median MIC value (MIC50) was 2.5 μM, while the MIC90 was 5 μM (range: 1.25–5.0 μM / geometric mean: 2.74 μM). Median MFC was 5.0 μM (range: 2.5->10 μM). MFC:MIC ratio was equal or lower than two for nine strains (60%), indicating that auranofin was fungicidal against some *H*. *capsulatum* strains located into different genetic clades.

**Table 1:** Antifungal susceptibility data of auranofin against 15 *Histoplasma capsulatum* strains from distinct genetic clades.

Strain	Genetic clade	Auranofin MIC ^a^ (μM / μg/mL)	Auranofin MFC ^b^ (μM)	MFC/MIC ratio
G217B	NAm2	1.25 / 0.84	10 / 6.72	8
G184A	Panama	5 / 3.36	5 / 3.36	1
39942	Panama	1.25 / 0.84	5 / 3.36	4
CÃO 4	RJ	5 / 3.36	10 / 6.72	2
IGS 4/5	RJ	5 /3.36	5 / 3.36	1
TI01	RJ	5 / 3.36	>10 / >6.72	>2
39439	RJ	1.25 / 0.84	2.5 / 1.68	2
IPEC 24_11	RJ	5 / 3.36	10 / 6.72	2
IPEC 28_11	RJ	2.5 / 1.68	>10 / >6.72	>4
CE 25/14	Northeast BR1	1.25 / 0.84	10 / 6.72	4
INI 02/16	LAmB1	5 /3.36	10 / 6.72	2
IPEC 11_12	LAmB1	2.5 1.68	5 / 3.36	2
EH394H	LamA1	5 / 3.36	5 / 3.36	1
EH53	LAmA	1.25 / 0.84	5 /3.36	4
20231	Unknown1	2.5 / 1.68	5 / 3.36	2

^a^ MIC: Minimal inhibitory concentration

^b^ MFC: Minimal fungicidal concentration

### 3.2 Auranofin presents synergism with amphotericin B against *H*. *capsulatum*

The initial checkerboard experiment conducted using the *H*. *capsulatum* G217B reference strain revealed indifferent interactions between auranofin and itraconazole or caspofungin (FICI values of 0.548 and 2.0, respectively). Conversely, the interaction between auranofin and amphotericin B exhibited synergy for this strain (FICI = 0.176). Subsequently, we performed checkerboard assays with auranofin and amphotericin B on five additional strains from distinct genotypes. Table 2 describes the checkerboard results of auranofin and amphotericin B for these six strains. Notably, this interaction demonstrated synergy in five strains and indifference in one.

**Table 2:** Checkerboard assay data of auranofin and amphotericin B against *Histoplasma capsulatum* strains from distinct genetic clades.

Strain	Genetic clade	MIC ^a^ (μM)			FICI ^d^	Interpretation
		AUR ^b^	AMB ^c^	AUR/AMB		
G217B	Nam2	1.25	0.125	0.156/0.008	0.1875	Synergism
G184A	Panama	5	0.03	0.625/0.008	0.375	Synergism
IGS 4/5	RJ	5	0.062	0.312/0.015	0.3125	Synergism
CE 25/14	Northeast BR1	2.5	0.03	0.312/0.008	0.375	Synergism
IPEC 11_12	LAmB	5	0.03	0.312/0.008	0.3125	Synergism
EH394H	LAmA1	5	0.015	5/0.008	1.5	Indifference

^a^ MIC: Minimal inhibitory concentration

^b^ AUR: Auranofin

^c^ AMB: Amphotericin B

^d^ FICI: fractional inhibitory concentration index

### 3.3 Auranofin changes *H*. *capsulatum* cellular size

**Fig 1:**
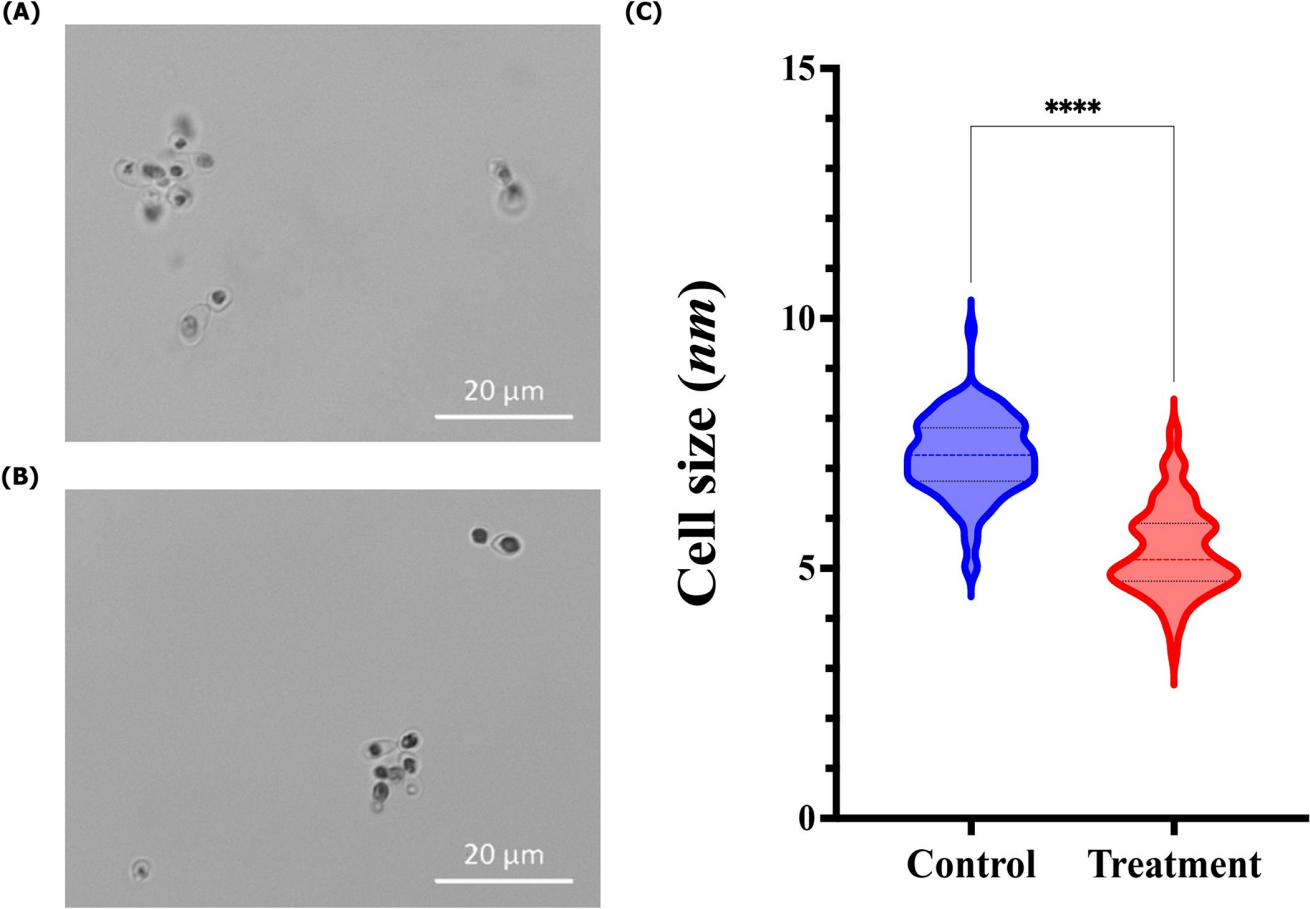
Influence of auranofin in the morphology of *Histoplasma capsulatum* G217B reference strain. (A) *H*. *capsulatum* yeast cells after 72 h incubation at 37°C in Ham´s F-12 drug-free medium; (B) *H*. *capsulatum* yeast cells after treatment with auranofin (0.625 μM) (Bars: 20 μm); (C) Comparison of the diameter size of *H*. *capsulatum* yeast cells, treated or not with auranofin. Optical microscopy images are representative of three independent experiments. The color line inside the violins represent the median diameter size and the black lines the interquartile ranges of 100 measurements performed in triplicate. **** P < 0.0001, Student´s t test.

Both auranofin-treated (Fig 1A) and -untreated (Fig 1B) *H*. *capsulatum* yeast cells presented a similar spherical to ovoid format. However, the diameter of *H*. *capsulatum* yeast cells treated with a subinhibitory concentration of auranofin showed a lower size when compared to the untreated control (P < 0.05, Fig 1C).

### 3.4 Auranofin regulates the expression of virulence-related genes

**Fig 2:**
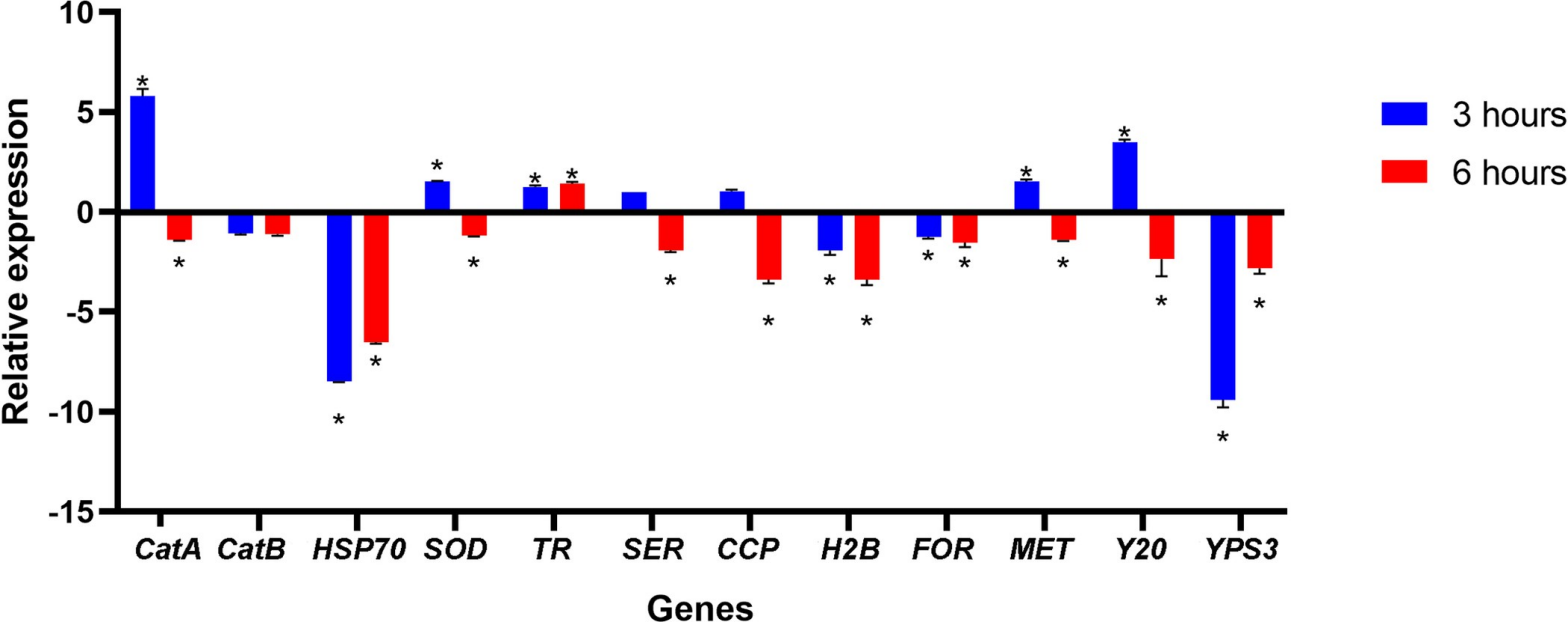
Influence of auranofin in the expression of some virulence-related genes by the *Histoplasma capsulatum* G217B reference strain. Results are presented as the mean and standard deviations derived from three independent experiments. The mean and standard deviations of the relative expression levels of each gene following three (blue bars) or six (red bars) hours of exposure to auranofin (0.625 μM) are illustrated. CatA: catalase A; CatB: catalase B; HSP70: heat shock protein 70 kDa; SOD: superoxide dismutase; TR: thioredoxin reductase; SER: serine proteinase; CCP: cytochrome C peroxidase; H2B: histone 2B; FOR: formamidase; MET: metallopeptidase; Y20: Y20 protein; YPS3: yeast phase specific protein 3. * P < 0.05, Student´s t test.

PCR efficiencies ranged between 90% and 110% for each of the primer pairs, indicating that all real time assays had similar good efficiencies, confirming the validation of the primers and the precision of experiments. These data are presented in S1 Table. Fig 2 presents the transcriptional levels of 12 genes related to fungal virulence. In general, the transcripts expression levels, except for catalase B, were significant altered in at least one of the two time points evaluated. After three hours of auranofin interaction, the genes coding for HSP70, serine proteinase, histone 2B, formamidase, and YPS3 were down-regulated, while the genes encoding catalase A, superoxide dismutase, thioredoxin reductase, metallopeptidase, and Y20 protein were up-regulated. On the other hand, after six hours of auranofin exposure, the gene encoding thioredoxin reductase was up-regulated, while all other genes, except for the catalase B gene, which was not affected by auranofin, were down-regulated.

### 3.5 Auranofin is effective against intracellular *H*. *capsulatum* yeast cells

**Fig 3:**
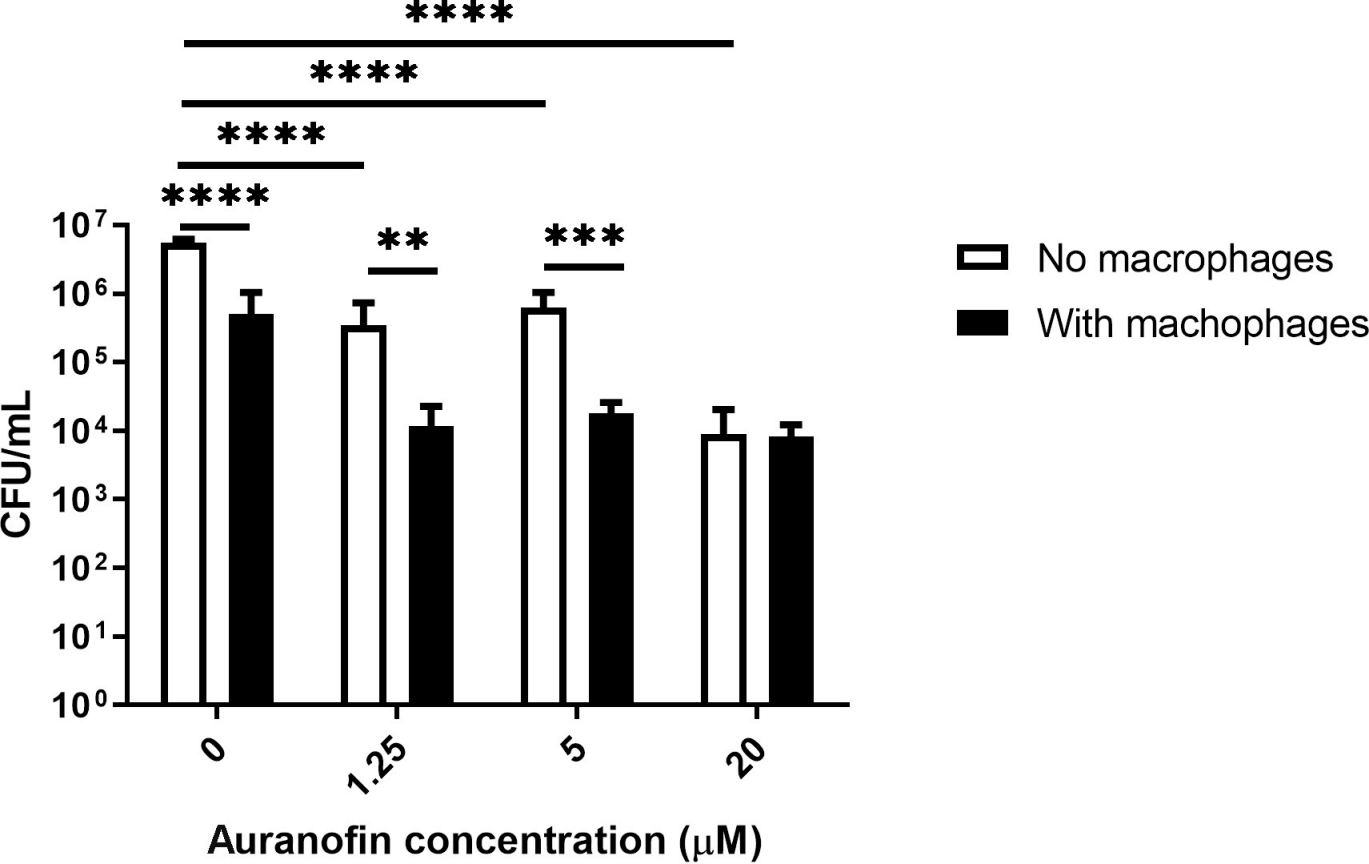
Antifungal activity of auranofin against intracellular *H*. *capsulatum* G217B reference strain. Results are presented as the mean and standard deviations derived from three independent experiments. The mean and standard deviations of the colony count units per milliliter (CFU/mL) of *H*. *capsulatum* planktonic (white bars) and intracellular (black bars) yeast cells after treatment with different auranofin concentrations (0, 0.125, 5, and 20 μM) are illustrated. ** P < 0.01, *** P < 0.001, **** P < 0.0001, Wilcoxon test.

Fig 3 presents the intracellular anti-*Histoplasma* activity of auranofin using a macrophage model. A reduced intracellular *H*. *capsulatum* CFU counts was observed as compared to the planktonic control, that is, same culture medium, but no macrophages (*P* < 0.0001). Auranofin at subinhibitory (1.25 μM / 0.84 μg/mL) and inhibitory concentrations (5 μM / 3.36 μg/mL) reduced *H*. *capsulatum* CFU counts as compared with the planktonic (*P* < 0.0001) and intracellular controls, without auranofin supplementation (*P* < 0.05). Moreover, intracellular *H*. *capsulatum* CFU counts were lower than planktonic *H*. *capsulatum* in the presence of subinhibitory and inhibitory auranofin concentrations (*P* < 0.05). High auranofin concentration (20 μM / 13.4 μg/mL) also reduced *H*. *capsulatum* CFU counts compared to the planktonic (*P* < 0.0001) and intracellular control (*P* < 0.05), but CFU counts of intracellular and planktonic *H*. *capsulatum* cells at this drug concentration were similar (*P* = 0.7077).

### 3.6 Auranofin is effective in an experimental model of *H*. *capsulatum* infection

**Fig 4:**
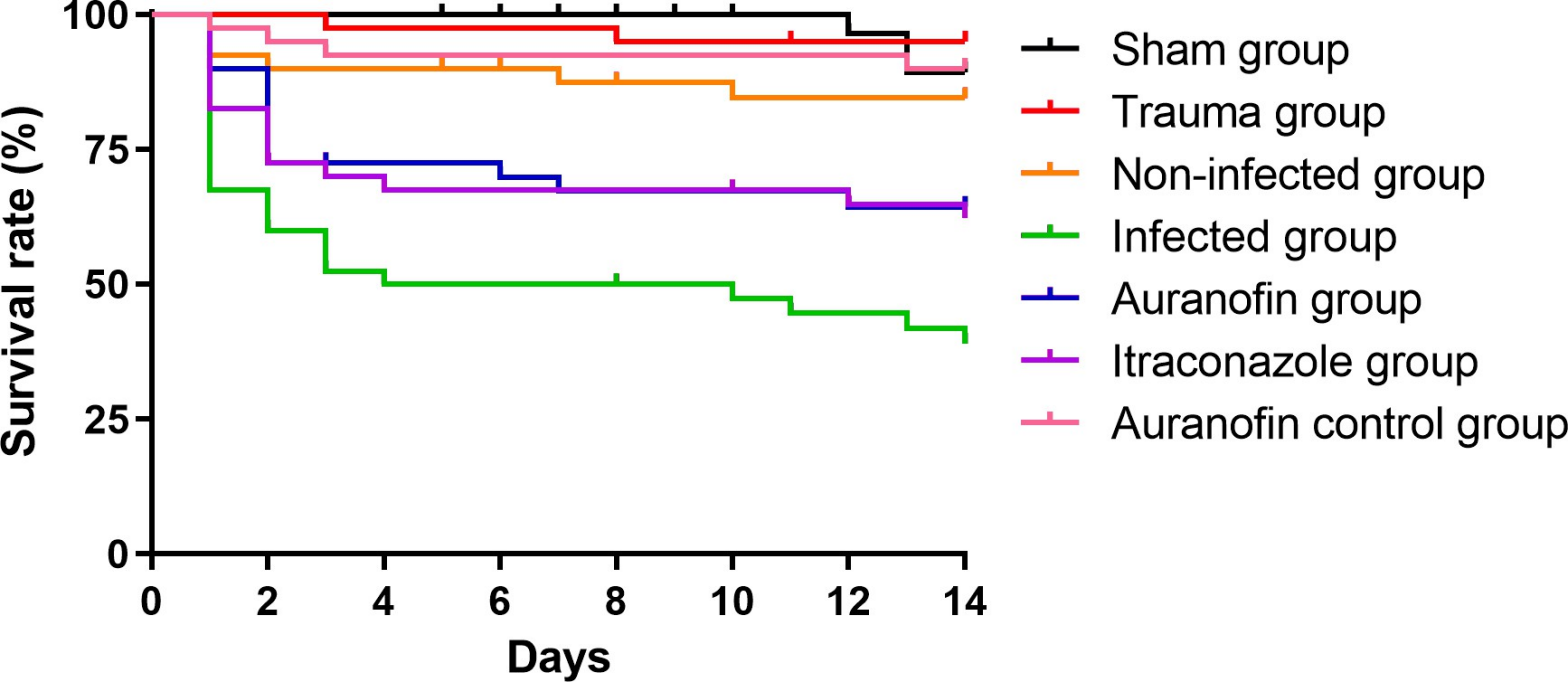
Antifungal activity of auranofin in the *Tenebrio molitor* infection model. Survival curves illustrate larvae infected with *Histoplasma capsulatum* G217B and treated with auranofin or itraconazole (5.7 mg/kg), represented by the blue and purple lines, respectively. Controls consisted in non-injected larvae (sham group, black line); larvae traumatized with the syringe (trauma group, red line); larvae injected with sterile PBS (non-infected group, orange line); infected with *H*. *capsulatum* and inoculated with PBS instead of auranofin or itraconazole (infected group, green line); and injected with PBS, instead of *H*. *capsulatum* cells and treated with auranofin 5.7 mg/kg (auranofin control group, pink line). Each curve represents a group of 30 larvae, monitored daily for survival for up to 14 days after infection. Larvae that converted to pupa were censored.

Fig 4 presents the survival curves of *T*. *molitor* larvae challenged with the reference *H*. *capsulatum* G217B strain. As expected, the sham, trauma, and non-infected groups presented more than 80% survival at the end of the experiment, as well as the auranofin control group that presented similar survival to the other control groups (*P* = 0.4265). Non-treated infected larvae presented 80% deaths, with a median survival of seven days. Animals treated with both itraconazole and auranofin were significantly protected, as compared to the infected group (*P =* 0.0407 and 0.0227, respectively). Moreover, survival curves of animals treated with itraconazole and auranofin did not present significant differences (*P* = 0.7969).

## 4 Discussion

Currently, drug repurposing is a promising subject into the drug discovery field, as it identifies new therapeutic opportunities for old drugs, already approved to treat another disease [41]. This strategy has gained importance to discover new drugs for neglected, rare or emerging diseases [42]. One of the strategies described to advance into the treatment of disseminated histoplasmosis is the drug repurposing approach, which would benefit individuals with histoplasmosis and other comorbidities [43]. Here, we present auranofin as a good candidate for the treatment of this life-threatening systemic mycosis. Besides anti-*Histoplasma* activity, this anti-rheumatic drug is also effective against other diseases that can occur in patients with histoplasmosis, such as neurotoxoplasmosis [3], drug-resistant bacterial infections [44], several types of cancer [45], candidiasis, and cryptococcosis [46], which improves the attractiveness towards auranofin repurposing.

The first evidence for the auranofin repurposing potential for histoplasmosis was its good *in vitro* activity against *H*. *capsulatum* strains of different genetic genotypes, such as NAm2, LAmA, LAmB1, Panama, RJ, Northeast BR1, and Unknown1. The MIC herein described, 1.25 to 5 μM (0.85 to 3.4 μg/mL), are similar to those found for other fungi. For instance, auranofin MICs against *Scedosporium* spp. ranged from 1 to 8 μg/mL [47], against black fungi that cause chromoblastomycosis from 1.25 to 2.5 μM [9], against *Candida* spp. from 0.25 to >16 μg/mL, and from 1 to 2 μg/mL against both *Cryptococcus neoformans* and *Blastomyces dermatitidis* [8]. Moreover, it has been described that auranofin at 32 μg/mL reduces the viability rate of mammalian cells by no more than 50%, indicating that auranofin toxicity is higher for *H*. *capsulatum* [48]. In addition to its isolated anti-*Histoplasma* activity, auranofin also demonstrated *in vitro* synergism with amphotericin B, a medication commonly employed in the treatment of histoplasmosis, among the majority of strains tested. This observation indicates a promising prospect for future therapeutic interventions against this mycosis. It is important to emphasize that the low bioabsorption of auranofin, estimated to be around 25% [45], underscores the need for adjustments in the formulation of this drug to effectively translate the results of this study in clinical practice.

Good candidates for drug repurposing against mycotic diseases should have a fungicidal, rather than a fungistatic nature [49]. In the present study, auranofin was fungicidal against 53.8% of the *H*. *capsulatum* strains tested, which is another advantage of this drug in the context of histoplasmosis treatment. Auranofin has also showed a fungicidal profile against *Phialophora verrucosa* and *Exophiala dermatitidis* [9].

Treatment of fungal cells with repurposed drugs may induce modifications in their cellular morphology [50]. For this reason, we investigated whether auranofin may induce this phenotypic change and it was observed that despite retaining the original shape, auranofin-treated cells were significantly smaller. This result was intriguing, as previous studies have described an alternative mechanism of action for auranofin in fungal cells involving the Mia40–Erv1 pathway. According to this model, auranofin induces a metabolic shift from respiration to fermentation without disrupting membrane potential or mitochondrial function [45]. However, yeast cells undergoing fermentation are typically larger than those undergoing respiration [51], This observation suggests that either the Mia40–Erv1 pathway is not involved in auranofin’s mechanism of action in *H*. *capsulatum*, or the relationship between cell size and metabolic state (fermentation versus respiration) in *H*. *capsulatum* differs from that observed in *S*. *cerevisiae*.

Cellular size is connected with virulence in some fungal species. For instance, smaller cells of *C*. *neoformans*, *Mucor circinelloides*, and *Paracoccidioides brasiliensis* are more likely to be phagocytosed and killed by macrophages [52–54]. To the best of our knowledge, there are no studies linking *H*. *capsulatum* virulence with its cellular size, but it is possible that a similar behavior occurs with this dimorphic fungus, thus auranofin may contribute to reduce *H*. *capsulatum* virulence. In our *in vitro* model of macrophage / *H*. *capsulatum* interaction, auranofin at inhibitory and subinhibitory concentrations enhanced macrophage killing of fungal yeast-like cells. This may have occurred by a direct action of auranofin in intracellular *H*. *capsulatum* cells or indirectly, by a reduction of yeast size, facilitating macrophage killing.

In fact, there are some studies reporting virulence reduction when pathogenic fungi are treated with compounds presenting antifungal activity [55–57]. To evaluate this issue, it was performed a qPCR assay targeting some genes associated with virulence in *H*. *capsulatum* or other fungal models. Two catalases were evaluated and only catalase A presented gene expression alterations in response to auranofin treatment. This is similar to what happens with transcript levels of this gene in response to cell morphology or oxidative stress [58]. Several genes related to the oxidative stress were down-regulated, especially after six hours of drug interaction. However, the thioredoxin reductase gene was the sole evaluated gene up-regulated on the two time points herein studied. In human cells, auranofin is a strong thioredoxin reductase enzymatic inhibitor [7]. The results of the current study do not allow us to conclude whether the same mechanism of action occurs in *H*. *capsulatum*. However, if this is indeed the case, the overexpression of this gene is likely a compensatory mechanism necessary because the existing enzymes are rendered non-functional as a result of auranofin action. In addition, the early up-regulation of catalase A, superoxide dismutase, and thioredoxin reductase suggests an oxidative stress mode of action of auranofin. Another interesting and strongly down-regulated gene was *YPS3*. This protein is located in cell surface and produced only by the pathogenic yeast-like form of the fungus, probably associated with fungal dissemination to some host organs, especially liver and spleen [59]. This is another encouraging result that drives the repurposing study of auranofin to treat histoplasmosis.[46]. A similar mechanism may occur in *H*. *capsulatum* yeast cells, which remains to be elucidated.

Finally, we used an invertebrate model to test the *in vivo* activity of auranofin. In last years, these animal models have gained importance in several science fields as a response to the public concern about the use of mammalian animals in research studies, with good success in several areas of biology and medicine [60]. However, some limitations of their use in drug discovery are the differences among invertebrate and human proteins, the absence of an adaptive immune response, and the lack of drug metabolism in insects [61]. In this study, auranofin showed to be as protective as itraconazole in the *T*. *molitor* larvae model. This model has gained relevance in medical mycology, including the antifungal study field, because this insect, which is susceptible to a broad range of pathogenic fungi, has a complex innate immunity, comprising cellular and humoral components [62]. Again, the auranofin activity in this experiment may be a direct result of the antifungal action of auranofin on *H*. *capsulatum* cells or an indirect result of the reduction of *H*. *capsulatum* virulence arsenal. Quantifying fungal CFU in infected animals would help clarify this issue. However, *H*. *capsulatum* is a slow-growing fungus, and recovering CFU of this species from larvae is extremely challenging due to the presence of contaminant fungi in the larvae microbiota, which grow faster than *H*. *capsulatum*. Future studies with mammalian hosts should address these hypotheses.

The repositioning of auranofin as an anti-*Histoplasma* drug holds significant relevance for public health. Originally developed as a gold compound for rheumatoid arthritis, auranofin emerges as a promising candidate for treating histoplasmosis, a fungal infection that can be life-threatening, particularly in immunocompromised individuals. Its potential repurposing offers a cost-effective and readily available therapeutic option, which is crucial given the high costs and limited accessibility of current antifungal treatments. By providing a viable alternative, auranofin could enhance treatment accessibility, reduce healthcare burdens, and improve patient outcomes in regions where histoplasmosis is prevalent, ultimately contributing to a more effective management of this serious infectious disease.

In addition to the limitations related to the animal model used for the in vivo study discussed above, there are other weaknesses in the current work: (i) we were unable to include H. capsulatum strains representing all known genotypes; (ii) there may be other genes with differential expression after auranofin treatment that were not evaluated in this study; and (iii) for most strains, the auranofin MIC was higher than the maximum plasma gold concentration following a daily administration of 6 mg of auranofin for one week, which is 1.584 μM [1]. This indicates that some modifications to this medication are necessary to adapt it for use in histoplasmosis patients.

In conclusion, auranofin seems to be a promising candidate for a repurposed drug to treat histoplasmosis, due to its fungicidal nature against most *H*. *capsulatum* strains and its potential to reduce *H*. *capsulatum* virulence.

## Supporting information

S1 Table qPCR primers, slope values, and efficiency of the *Histoplasma capsulatum* G217B primers used in this study. (XLSX)

## References

[1] Yamashita M. Auranofin: Past to Present, and repurposing. Int Immunopharmacol. 2021;101: 108272. doi: 10.1016/j.intimp.2021.108272 34731781

[2] Harbut MB, Vilchèze C, Luo X, Hensler ME, Guo H, Yang B, et al. Auranofin exerts broad-spectrum bactericidal activities by targeting thiol-redox homeostasis. Proc Natl Acad Sci U S A. 2015;112: 4453–4458. doi: 10.1073/pnas.1504022112 25831516 PMC4394260

[3] Abou-El-Naga IF, Mogahed NMFH. Repurposing auranofin for treatment of Experimental Cerebral Toxoplasmosis. Acta Parasitol. 2021;66: 827–836. doi: 10.1007/s11686-021-00337-z 33555553

[4] Abdalbari FH, Telleria CM. The gold complex auranofin: new perspectives for cancer therapy. Discov Oncol. 2021;12: 42. doi: 10.1007/s12672-021-00439-0 35201489 PMC8777575

[5] Sharma N, Singh A, Sharma R, Kumar A. Repurposing of Auranofin Against Bacterial Infections: An In silico and In vitro Study. Curr Comput Aided Drug Des. 2021;17: 687–701. doi: 10.2174/1386207323666200717155640 32679020

[6] Feng L, Pomel S, Latre de Late P, Taravaud A, Loiseau PM, Maes L, et al. Repurposing Auranofin and Evaluation of a New Gold(I) Compound for the Search of Treatment of Human and Cattle Parasitic Diseases: From Protozoa to Helminth Infections. Molecules. 2020;25: E5075. doi: 10.3390/molecules25215075 33139647 PMC7663263

[7] Sonzogni-Desautels K, Ndao M. Will Auranofin Become a Golden New Treatment Against COVID-19? Front Immunol. 2021;12: 683694. doi: 10.3389/fimmu.2021.683694 34630379 PMC8492993

[8] Wiederhold NP, Patterson TF, Srinivasan A, Chaturvedi AK, Fothergill AW, Wormley FL, et al. Repurposing auranofin as an antifungal: In vitro activity against a variety of medically important fungi. Virulence. 2017;8: 138–142. doi: 10.1080/21505594.2016.1196301 27268469 PMC5354159

[9] Coelho RA, Joffe LS, Alves GM, Figueiredo-Carvalho MHG, Brito-Santos F, Amaral ACF, et al. A screening of the MMV Pathogen Box® reveals new potential antifungal drugs against the etiologic agents of chromoblastomycosis. PLoS One. 2020;15: e0229630. doi: 10.1371/journal.pone.0229630 32401759 PMC7219733

[10] Gamberi T, Fiaschi T, Modesti A, Massai L, Messori L, Balzi M, et al. Evidence that the antiproliferative effects of auranofin in *Saccharomyces cerevisiae* arise from inhibition of mitochondrial respiration. Int J Biochem Cell Biol. 2015;65: 61–71. doi: 10.1016/j.biocel.2015.05.016 26024642

[11] Guerra BT, Almeida-Silva F, Almeida-Paes R, Basso RP, Bernardes JPRA, Almeida MA, et al. Histoplasmosis Outbreaks in Brazil: Lessons to Learn About Preventing Exposure. Mycopathologia. 2020;185: 881–892. doi: 10.1007/s11046-019-00389-w 31845177

[12] Capone D, Wanke B, Monteiro PC, Lazéra MS, de Noronha Andrade G, do Valle AC, et al. Chronic pulmonary histoplasmosis in the State of Rio de Janeiro, Brazil. Mycopathologia. 1999;145: 75–79. doi: 10.1023/a:1007016414833 10598067

[13] Nacher M, Valdes A, Adenis A, Blaizot R, Abboud P, Demar M, et al. Disseminated Histoplasmosis in HIV-Infected Patients: A Description of 34 Years of Clinical and Therapeutic Practice. J Fungi (Basel). 2020;6: E164. doi: 10.3390/jof6030164 32906589 PMC7557823

[14] Mittal J, Ponce MG, Gendlina I, Nosanchuk JD. *Histoplasma capsulatum*: Mechanisms for Pathogenesis. Curr Top Microbiol Immunol. 2019;422: 157–191. doi: 10.1007/82_2018_114 30043340 PMC7212190

[15] Nacher M, Couppié P, Epelboin L, Djossou F, Demar M, Adenis A. Disseminated Histoplasmosis: Fighting a neglected killer of patients with advanced HIV disease in Latin America. PLoS Pathog. 2020;16: e1008449. doi: 10.1371/journal.ppat.1008449 32407383 PMC7224450

[16] Nacher M, Leitao TS, Gómez BL, Couppié P, Adenis A, Damasceno L, et al. The Fight against HIV-Associated Disseminated Histoplasmosis in the Americas: Unfolding the Different Stories of Four Centers. J Fungi. 2019;5: 51. doi: 10.3390/jof5020051 31212897 PMC6617033

[17] Rajasingham R, Medina N, Mousquer GT, Caceres DH, Jordan A, Nacher M, et al. Cost-effectiveness evaluation of routine histoplasmosis screening among people living with advanced HIV disease in Latin America and the Caribbean. PLOS Glob Public Health. 2023;3: e0001861. doi: 10.1371/journal.pgph.0001861 37582115 PMC10427011

[18] Sanguinetti M, Posteraro B, Beigelman-Aubry C, Lamoth F, Dunet V, Slavin M, et al. Diagnosis and treatment of invasive fungal infections: looking ahead. J Antimicrob Chemother. 2019;74: ii27–ii37. doi: 10.1093/jac/dkz041 31222314

[19] Thompson GR, Le T, Chindamporn A, Kauffman CA, Alastruey-Izquierdo A, Ampel NM, et al. Global guideline for the diagnosis and management of the endemic mycoses: an initiative of the European Confederation of Medical Mycology in cooperation with the International Society for Human and Animal Mycology. Lancet Infect Dis. 2021;21: e364–e374. doi: 10.1016/S1473-3099(21)00191-2 34364529 PMC9450022

[20] Suberviola B. Seguridad clínica de la anfotericina B liposomal. Rev Iberoam Micol. 2021;38: 56–60. doi: 10.1016/j.riam.2021.02.001 33994103

[21] Wang Y, Lipner SR. Retrospective analysis of adverse events with systemic onychomycosis medications reported to the United States Food and Drug Administration. J Dermatolog Treat. 2021;32: 783–787. doi: 10.1080/09546634.2019.1708242 31865826

[22] Hoenigl M, Sprute R, Egger M, Arastehfar A, Cornely OA, Krause R, et al. The Antifungal Pipeline: Fosmanogepix, Ibrexafungerp, Olorofim, Opelconazole, and Rezafungin. Drugs. 2021;81: 1703–1729. doi: 10.1007/s40265-021-01611-0 34626339 PMC8501344

[23] Mittal N, Mittal R. Repurposing old molecules for new indications: Defining pillars of success from lessons in the past. European Journal of Pharmacology. 2021;912: 174569. doi: 10.1016/j.ejphar.2021.174569 34653378

[24] Almeida-Paes R, Frases S. Repurposing drugs for fungal infections: advantages and limitations. Future Microbiol. 2023;18: 1013–1016. doi: 10.2217/fmb-2023-0108 37721174

[25] Kasuga T, White TJ, Koenig G, McEwen J, Restrepo A, Castañeda E, et al. Phylogeography of the fungal pathogen Histoplasma capsulatum. Mol Ecol. 2003;12: 3383–3401. doi: 10.1046/j.1365-294x.2003.01995.x 14629354

[26] Vite-Garín T, Estrada-Bárcenas DA, Cifuentes J, Taylor ML. The importance of molecular analyses for understanding the genetic diversity of Histoplasma capsulatum: an overview. Rev Iberoam Micol. 2014;31: 11–15. doi: 10.1016/j.riam.2013.09.013 24252830

[27] Damasceno LS, Teixeira M de M, Barker BM, Almeida MA, Muniz M de M, Pizzini CV, et al. Novel clinical and dual infection by *Histoplasma capsulatum* genotypes in HIV patients from Northeastern, Brazil. Sci Rep. 2019;9: 11789. doi: 10.1038/s41598-019-48111-6 31409874 PMC6692370

[28] Almeida-Silva F, de Melo Teixeira M, Matute DR, de Faria Ferreira M, Barker BM, Almeida-Paes R, et al. Genomic Diversity Analysis Reveals a Strong Population Structure in *Histoplasma capsulatum* LAmA (*Histoplasma suramericanum*). J Fungi. 2021;7: 865. doi: 10.3390/jof7100865 34682286 PMC8540737

[29] Fressatti R, Dias-Siqueira VL, Svidzinski TIE, Herrero F, Kemmelmeier C. A medium for inducing conversion of *Histoplasma capsulatum* var. *capsulatum* into its yeast-like form. Mem Inst Oswaldo Cruz. 1992;87: 53–58. doi: 10.1590/S0074-02761992000100010 1308555

[30] EUCAST. EUCAST definitive document EDef 7.1: method for the determination of broth dilution MICs of antifungal agents for fermentative yeasts. Clin Microbiol Infect. 2008;14: 398–405. doi: 10.1111/j.1469-0691.2007.01935.x 18190574

[31] Franconi I, Lupetti A. In Vitro Susceptibility Tests in the Context of Antifungal Resistance: Beyond Minimum Inhibitory Concentration in *Candida* spp. J Fungi. 2023;9: 1188. doi: 10.3390/jof9121188 38132789 PMC10744879

[32] Dannaoui E, Afeltra J, Meis JFGM, Verweij PE, Eurofung Network. In vitro susceptibilities of zygomycetes to combinations of antimicrobial agents. Antimicrob Agents Chemother. 2002;46: 2708–2711. doi: 10.1128/AAC.46.8.2708-2711.2002 12121963 PMC127328

[33] Odds FC. Synergy, antagonism, and what the chequerboard puts between them. J Antimicrob Chemother. 2003;52: 1. doi: 10.1093/jac/dkg301 12805255

[34] Almeida MA, Baeza LC, Almeida-Paes R, Bailão AM, Borges CL, Guimarães AJ, et al. Comparative Proteomic Analysis of *Histoplasma capsulatum* Yeast and Mycelium Reveals Differential Metabolic Shifts and Cell Wall Remodeling Processes in the Different Morphotypes. Front Microbiol. 2021;12: 640931. doi: 10.3389/fmicb.2021.640931 34177824 PMC8226243

[35] Edwards JA, Chen C, Kemski MM, Hu J, Mitchell TK, Rappleye CA. *Histoplasma* yeast and mycelial transcriptomes reveal pathogenic-phase and lineage-specific gene expression profiles. BMC Genomics. 2013;14: 695. doi: 10.1186/1471-2164-14-695 24112604 PMC3852720

[36] Joehnk B, Ali N, Voorhies M, Walcott K, Sil A. Recyclable CRISPR/Cas9-mediated gene disruption and deletions in *Histoplasma*. mSphere. 2023;8: e0037023. doi: 10.1128/msphere.00370-23 37819140 PMC10732100

[37] Bookout AL, Cummins CL, Mangelsdorf DJ, Pesola JM, Kramer MF. High-throughput real-time quantitative reverse transcription PCR. Curr Protoc Mol Biol. 2006;Chapter 15: Unit 15.8. doi: 10.1002/0471142727.mb1508s73 18265376

[38] Parente-Rocha JA, Parente AFA, Baeza LC, Bonfim SMRC, Hernandez O, McEwen JG, et al. Macrophage Interaction with *Paracoccidioides brasiliensis* Yeast Cells Modulates Fungal Metabolism and Generates a Response to Oxidative Stress. PLoS One. 2015;10: e0137619. doi: 10.1371/journal.pone.0137619 26360774 PMC4567264

[39] Lozoya-Pérez NE, García-Carnero LC, Martínez-Álvarez JA, Martínez-Duncker I, Mora-Montes HM. Tenebrio molitor as an Alternative Model to Analyze the *Sporothrix* Species Virulence. Infect Drug Resist. 2021;Volume 14: 2059–2072. doi: 10.2147/IDR.S312553 34113132 PMC8184153

[40] Lim W, Konings M, Parel F, Eadie K, Strepis N, Fahal A, et al. Inhibiting DHN- and DOPA-melanin biosynthesis pathway increased the therapeutic value of itraconazole in *Madurella mycetomatis* infected *Galleria mellonella*. Med Mycol. 2022;60: myac003. doi: 10.1093/mmy/myac003 35064672 PMC9295015

[41] Doan TL, Pollastri M, Walters MA, Georg GI. The Future of Drug Repositioning. Annual Reports in Medicinal Chemistry. Elsevier; 2011. pp. 385–401. doi: 10.1016/B978-0-12-386009-5.00004–7

[42] Talevi A. Drug Repurposing. Comprehensive Pharmacology. Elsevier; 2022. pp. 813–824. doi: 10.1016/B978-0-12-820472-6.00108–0

[43] Pasqualotto AC, Queiroz-Telles F, Chebabo A, Leitao TMJS, Falci DR, Xavier MO, et al. The “Histoplasmosis Porto Alegre manifesto”-Addressing disseminated histoplasmosis in AIDS. PLoS Negl Trop Dis. 2023;17: e0010960. doi: 10.1371/journal.pntd.0010960 36602963 PMC9815569

[44] Mohammad H, Abutaleb NS, Seleem MN. Auranofin Rapidly Eradicates Methicillin-resistant *Staphylococcus aureus* (MRSA) in an Infected Pressure Ulcer Mouse Model. Sci Rep. 2020;10: 7251. doi: 10.1038/s41598-020-64352-2 32350417 PMC7190694

[45] Onodera T, Momose I, Kawada M. Potential Anticancer Activity of Auranofin. Chem Pharm Bull (Tokyo). 2019;67: 186–191. doi: 10.1248/cpb.c18-00767 30827998

[46] Thangamani S, Maland M, Mohammad H, Pascuzzi PE, Avramova L, Koehler CM, et al. Repurposing Approach Identifies Auranofin with Broad Spectrum Antifungal Activity That Targets Mia40-Erv1 Pathway. Front Cell Infect Microbiol. 2017;7: 4. doi: 10.3389/fcimb.2017.00004 28149831 PMC5241286

[47] Yaakoub H, Staerck C, Mina S, Godon C, Fleury M, Bouchara J-P, et al. Repurposing of auranofin and honokiol as antifungals against *Scedosporium* species and the related fungus Lomentospora prolificans. Virulence. 2021;12: 1076–1090. doi: 10.1080/21505594.2021.1909266 33825667 PMC8032236

[48] Lu H, Lu W, Zhu Y, Wang C, Shi L, Li X, et al. Auranofin Has Advantages over First-Line Drugs in the Treatment of Severe *Streptococcus suis* Infections. Antibiotics. 2020;10: 26. doi: 10.3390/antibiotics10010026 33396878 PMC7823847

[49] Wong SSW, Samaranayake LP, Seneviratne CJ. In pursuit of the ideal antifungal agent for *Candida* infections: high-throughput screening of small molecules. Drug Discov Today. 2014;19: 1721–1730. doi: 10.1016/j.drudis.2014.06.009 24952336

[50] Almeida-Paes R, de Andrade IB, Ramos MLM, Rodrigues MV de A, do Nascimento VA, Bernardes-Engemann AR, et al. Medicines for Malaria Venture COVID Box: a source for repurposing drugs with antifungal activity against human pathogenic fungi. Mem Inst Oswaldo Cruz. 2021;116: e210207. doi: 10.1590/0074-02760210207 34755820 PMC8577065

[51] Maslanka R, Zadrag-Tecza R. Reproductive Potential of Yeast Cells Depends on Overall Action of Interconnected Changes in Central Carbon Metabolism, Cellular Biosynthetic Capacity, and Proteostasis. IJMS. 2020;21: 7313. doi: 10.3390/ijms21197313 33022992 PMC7582853

[52] Almeida AJ, Cunha C, Carmona JA, Sampaio-Marques B, Carvalho A, Malavazi I, et al. Cdc42p controls yeast-cell shape and virulence of *Paracoccidioides brasiliensis*. Fungal Genet Biol. 2009;46: 919–926. doi: 10.1016/j.fgb.2009.08.004 19686860

[53] Li CH, Cervantes M, Springer DJ, Boekhout T, Ruiz-Vazquez RM, Torres-Martinez SR, et al. Sporangiospore size dimorphism is linked to virulence of *Mucor circinelloides*. PLoS Pathog. 2011;7: e1002086. doi: 10.1371/journal.ppat.1002086 21698218 PMC3116813

[54] Crabtree JN, Okagaki LH, Wiesner DL, Strain AK, Nielsen JN, Nielsen K. Titan cell production enhances the virulence of *Cryptococcus neoformans*. Infect Immun. 2012;80: 3776–3785. doi: 10.1128/IAI.00507-12 22890995 PMC3486048

[55] Man A, Mare A-D, Mares M, Ruta F, Pribac M, Maier A-C, et al. Antifungal and anti-virulence activity of six essential oils against important *Candida* species—a preliminary study. Future Microbiol. 2022;17: 737–753. doi: 10.2217/fmb-2021-0296 35531749

[56] Huang L, Zhang J, Song T, Yuan L, Zhou J, Yin H, et al. Antifungal curcumin promotes chitin accumulation associated with decreased virulence of *Sporothrix schenckii*. Int Immunopharmacol. 2016;34: 263–270. doi: 10.1016/j.intimp.2016.03.010 26995026

[57] Samie S, Trollope KM, Joubert L-M, Makunga NP, Volschenk H. The antifungal and *Cryptococcus neoformans* virulence attenuating activity of *Pelargonium sidoides* extracts. J Ethnopharmacol. 2019;235: 122–132. doi: 10.1016/j.jep.2019.02.008 30738119

[58] Johnson CH, Klotz MG, York JL, Kruft V, McEwen JE. Redundancy, phylogeny and differential expression of *Histoplasma capsulatum* catalases. Microbiology. 2002;148: 1129–1142. doi: 10.1099/00221287-148-4-1129 11932457

[59] Bohse ML, Woods JP. RNA interference-mediated silencing of the YPS3 gene of *Histoplasma capsulatum* reveals virulence defects. Infect Immun. 2007;75: 2811–2817. doi: 10.1128/IAI.00304-07 17403872 PMC1932869

[60] Wilson-Sanders SE. Invertebrate models for biomedical research, testing, and education. ILAR J. 2011;52: 126–152. doi: 10.1093/ilar.52.2.126 21709307

[61] Ségalat L. Invertebrate Animal Models of Diseases as Screening Tools in Drug Discovery. ACS Chem Biol. 2007;2: 231–236. doi: 10.1021/cb700009m 17455900

[62] Canteri de Souza P, Custódio Caloni C, Wilson D, Sergio Almeida R. An Invertebrate Host to Study Fungal Infections, Mycotoxins and Antifungal Drugs: *Tenebrio molitor*. J Fungi. 2018;4: 125. doi: 10.3390/jof4040125 30424549 PMC6308941

